# Clinical and radiographic outcomes of extra-short implants (≤ 6 mm) in the posterior atrophic jaws: a retrospective cohort study

**DOI:** 10.1186/s40729-025-00592-z

**Published:** 2025-01-20

**Authors:** Stefano Sivolella, Stefano Giovannini, Joana Berberi, Michele Stocchero, Giulia Brunello

**Affiliations:** 1 Department of Neurosciences, School of Dentistry, University of Padua, Padua, Italy; 2 Department of Biomedical, Surgical and Dental Sciences, University of Milan, Milan, Italy; 3 Department of Oral & Maxillofacial Surgery and Oral Medicine, Faculty of Odontology, Malmö University, Malmö, Sweden; 4 Department of Oral Surgery, University Hospital of Düsseldorf, Düsseldorf, Germany; 5 Department of Orthodontics and Dentofacial Orthopedics, Charité - Universitätsmedizin Berlin, corporate member of Freie Universität Berlin and Humboldt-Universität zu Berlin, Berlin, Germany

**Keywords:** Extra-short implants, Implant survival, Marginal bone loss, Immediate loading, Platform switching

## Abstract

**Objective:**

This study aimed at investigating implant survival rate and marginal bone loss (MBL) around extra-short implants. The impact of the loading protocol and of the use of an intermediate abutment was also evaluated, to explore possible differences in terms of the outcome measures.

**Materials and methods:**

Patients with single or multiple mandibular or maxillary posterior edentulism rehabilitated using extra-short 5–6 mm long implants were included. Different prosthetic protocols were used. Clinical and radiological follow-up was 5 years. The outcomes measures were implant survival and MBL.

**Results:**

The analysis included 56 implants placed in 34 adults (12 males and 22 females; mean age 60 years, SD 11). Six implants failed during a median follow-up of 5 years and 4 of them were recorded in one patient at 2-year follow-up. The 5-year implant survival was 89% overall (87% in conventional and 94% in immediate loading). At univariate analysis, during follow-up immediate loading was associated with higher MBL (mean variation 0.21 mm, 95%CI 0.01 to 0.40; *p* = 0.02), while intermediate abutment was associated with lower MBL (mean variation -0.23 mm, 95%CI -0.39 to -0.09; *p* = 0.003). Multivariable analysis confirmed that immediate loading was associated with higher MBL.

**Conclusion:**

Within its limitations, this study showed that extra-short implants under immediate loading conditions can be a reliable solution. The application of horizontal and vertical platform switching with the use of intermediate abutments seems to be able to contribute to the reduction of MBL.

**Graphical Abstract:**

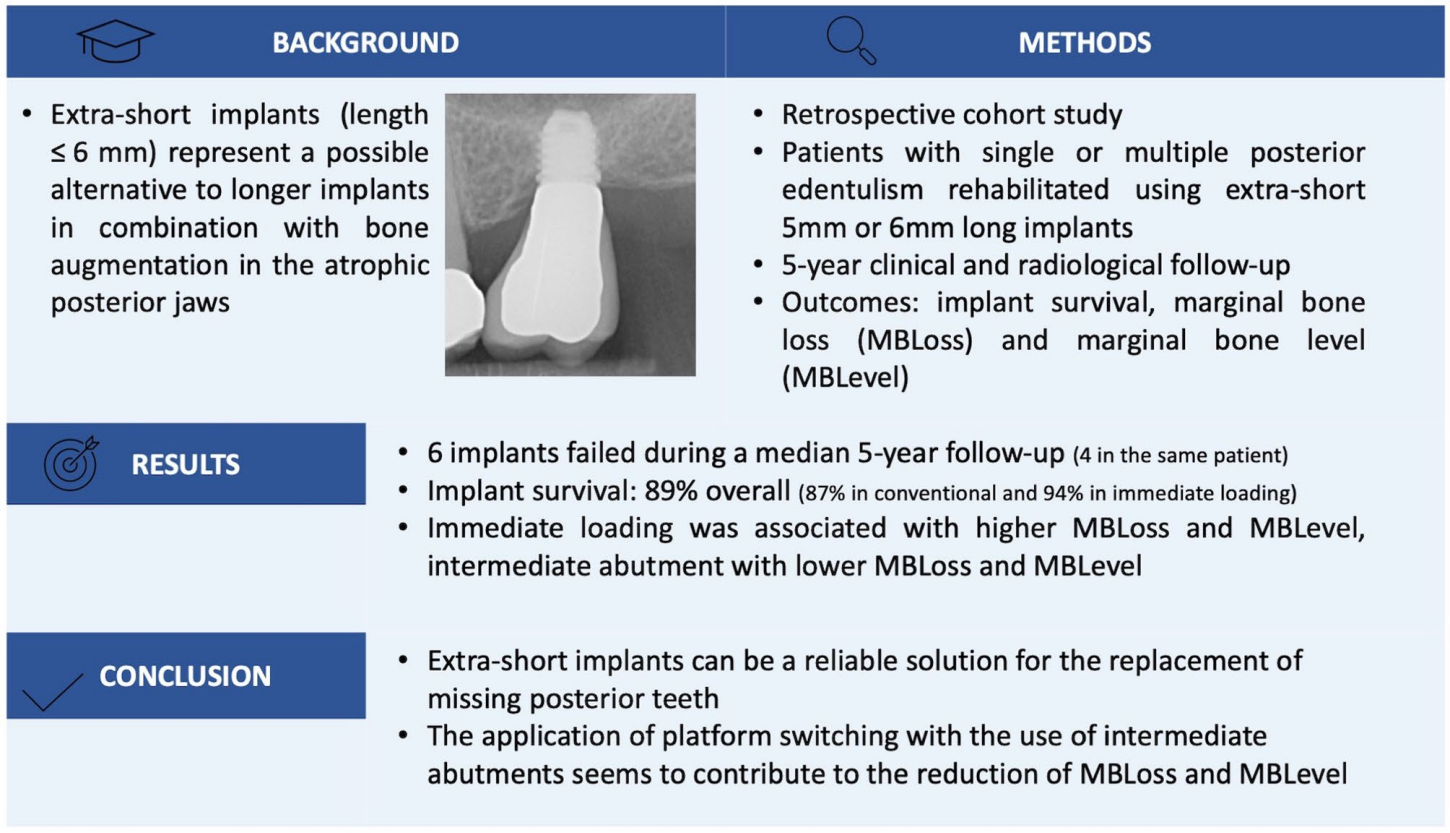

**Supplementary Information:**

The online version contains supplementary material available at 10.1186/s40729-025-00592-z.

## Introduction

Dental implants have been demonstrated to be reliable solutions for the replacement of missing teeth owing to their high long-term survival rates [1, 2]. Extra-short implants (length ≤ 6 mm) represent a possible alternative to longer implants in combination with bone augmentation in the atrophic posterior jaws [3, 4]. They allow to reduce the number and invasiveness of treatment procedures, the operative time, the morbidity, as well as the overall treatment cost [5–7].

Several studies have shown survival rates of short implants to be comparable to standard implants [8–13]. A 5-year follow-up multicenter randomized controlled trial (RCT) showed no difference between 6 mm and 11 mm implants in terms of marginal bone level change, implant/prosthetic survival rate, and biological or technical complications occurrence, concluding that short implants can be a reliable option in the mandible [14]. A 5-year survival rate of 96.0% for 6 mm short implants vs. 98.9% for 11 mm long implants has been reported, without any significant difference between the two groups [15]. A recent systematic review and meta-analysis of RCTs with follow-up ranging from 1 to 10 years compared standard dental implants to short implants [16]. The latter presented less peri-implant bone loss and a reduced risk of biological complications, despite no significant differences in implant failure. Short implants have exhibited similar clinical and radiographic results to standard implants associated with sinus floor elevation [17–21]. In a 10-year retrospective study, partial fixed dental prostheses fabricated with a milled fiber-reinforced composite framework supported by short or extra-short implants, were investigated [22]. This study showed high survival rate (95.9%) and few complications (10.2%) up to 10 years. A meta-analysis on the survival rates of short dental implants (≤ 6 mm) compared with implants longer than 6 mm in posterior jaw areas concluded that short implants have higher variability and lower predictability in survival rates compared to longer implants after periods of 1 to 5 years in function [23]. The mean survival rate was 96% for short and 98% for longer implants. The risk ratio (RR) for short implant failure compared to longer implants was 1.29 (95% CI: 0.67, 2.50, *p* = 0.45), demonstrating that short implants (≤ 6 mm) had a 29% higher risk of failure to implants longer than 6 mm. Nevertheless, prosthesis survival for short and longer implants following a period of 1 to 5 years was similar (98.6% and 99.5%, respectively). A recent umbrella review with meta-analysis suggests that the use of short implants could decrease implant failure, marginal bone loss, and biological complications, and increase patient satisfaction [24].

Immediately loaded short implants have been successfully utilized in single- and multiple-unit fixed dental prostheses [10, 25–30].

Marginal bone loss is a critical event related to implant survival especially for short implants, due to their reduced length. Yet, it has been reported that the application of platform switching can reduce marginal bone remodelling [10]. Peri-implant bone remodelling around 8.5 mm long dental implants has been demonstrated to be positively influenced by the platform switching concept in the short term [31]. However, at five-year follow-up, in the same cohort of patients, the inter-proximal bone loss in presence of platform switching was comparable to the bone loss in the control group [32]. Horizontal platform switching can also be obtained on wide-diameter, external-hex-connection dental implants using prosthetic components characterized by narrower diameter [33, 34]. A similar concept can be developed vertically using intermediate abutments, whose presence is supposed to minimize crestal bone loss and optimize the formation and maintenance of healthy supracrestal tissue [35, 36]. The influence of platform switching, in combination or not with immediate loading, on peri-implant bone remodelling around large diameter extra-short implants remains to be clarified. The present retrospective study aimed at investigating implant survival rate, marginal bone loss (MBLoss) and marginal bone level (MBLevel) around extra-short 5–6 mm long implants. The impact of the loading protocol and of the use of an intermediate abutment on MBLoss and MBLevel was also evaluated, to explore possible differences in such strata.

## Materials and methods

This retrospective cohort study included patients who were treated with extra-short implants (5 mm and 6 mm length) for the replacement of at least one molar or premolar associated with maxillary or mandibular alveolar bone atrophy. This study was conducted in accordance with the Helsinki declaration as revised in 2013. All patients involved provided their consent prior to inclusion in the study. Ethics approval was obtained from the Hospital of Padova Ethic Board (Protocol Nr. 4376/AO/17). This study was reported according to the STROBE (Strengthening the Reporting of Observational studies in Epidemiology) guidelines [37].

All clinical and radiographical records between September 2013 and July 2022 were screened.

Patients were selected based on the following inclusion criteria: age ≥ 18 years old; ASA (American Society of Anaesthesiologists) classes 1 or 2; absence of one or more maxillary or mandibular molar and premolar associated with alveolar bone atrophy (available bone height between 6 mm and 8 mm, width at least 8 mm); availability of a periapical radiograph at baseline and every year during the follow-up. Exclusion criteria were: untreated or active periodontal disease; need for regenerative techniques; previous or ongoing chemotherapy or local radiotherapy; use of antiresorptive drugs.

The following data were recorded: demographic data (gender, age at the time of surgery); date of surgery; location of the implant; implant features (diameter, length); submerged healing (y/n); conventional or immediate loading; intermediate abutment (y/n); crown/implant ratio based on intraoral radiograph measurements (C/I); splinted (y/n); cantilever (y/n).

### Surgical procedures

Preliminary radiographic assessment included preoperative panoramic radiograph and cone beam computer tomography (CBCT) scan. All surgical procedures were conducted by a single operator (S.S.) under local anaesthesia. External hexagon implants having a diameter of 5–6 mm and a length of 5–6 mm (T3^®^ Super Short 3i, Zimmer Biomet, Palm Beach Gardens, FL, USA) were placed according to the manufacturer guidelines. When used, the cover screw always had a diameter of 4.1 mm.

### Prosthetic procedures

Loading protocols could include immediate or conventional loading (i.e. ≥2 months after implant placement). In case of immediate loading, a temporary abutment with prefabricated non-functionalized resin interim restoration was applied. In case of conventional loading, either a one-stage or a two-stage approach was selected. An intermediate abutment (Low Profile Abutment, Zimmer Biomet) was positioned depending on the clinical conditions. The implants were finally restored with screw-retained metal ceramic single- or multi-unit prostheses.

Regardless of the prosthetic protocol applied, healing screws, Low Profile and prosthetic abutments always had a diameter of 4.1 mm.

### Outcomes

The outcomes considered were implant survival rate, marginal bone loss (MBLoss) and marginal bone level (MBLevel) around implants. Implant survival was defined as the presence of the implants in the mouth at the time of examination, regardless of the state of the prosthesis or patient satisfaction. Survival time was defined as the time from implant insertion to implant removal or to last follow-up for surviving implants. Implant failure was defined as removal of an implant due to loss of osteointegration, spontaneous loss or mechanical issues.

MBLoss was evaluated on periapical radiographs (Fullsize CCD sensor, Sirona Heliodent DS X-ray source, Dentsply Sirona Inc., Charlotte, NC, US) routinely taken using long cone paralleling technique and an X-ray holder immediately after implant placement, at loading (baseline), at 1 year, and at annual follow-up appointments if available. Each radiographic image was evaluated by means of a specific software (ImageJ, National Institutes of Health, Bethesda, MD, USA). The known implant diameter (5–6 mm) was used to calibrate all radiographs for measurements and MBLoss was calculated. A line perpendicular to the implant axes connecting the mesial and distal edges of the implant shoulder (IS) was drawn and used as landmark. When assessing mesial and distal MBLoss, every value that showed a marginal bone over or at the implant shoulder was considered as zero. Bone loss was defined by negative values starting from the implant shoulder (Supplementary Fig. 1). To evaluate bone gain and bone resorption also taking into account the initial implant position, MBLevel was calculated as well, assigning a positive value when the bone crest was coronal to the IS, whereas a negative value was assigned when the bone crest was apical to the IS (Supplementary Fig. 2). All radiographic measurements were taken by one experienced examiner (S.G.) who was not involved in the treatment process.

The following biological and technical/mechanical complications were recorded by drawing information from medical records and review of available radiographs: mucositis and peri-implantitis, as defined in Berglundh et al. [38], implant fracture, fracture of the prosthetic components, veneer chipping, prosthetic screw loosening/fracture, any other possible complications found.

### Statistical analysis

Continuous data were summarized as mean and standard deviation (SD) or median and interquartile range (IQR). To assess the reproducibility of marginal bone level measurements, 13 measurements were obtained by the same operator twice, giving at least one week from the first and the latter, and intraclass correlation coefficient (ICC) was calculated. This number (*n* = 13) was estimated to have an 80% chance of detecting, as significant at the 5% level, an ICC > 80%. Implant survival curves were calculated using the Kaplan-Meier method. The investigation of factors associated with MBLoss and MBLevel during follow-up was conducted using linear mixed effect models, including implant and patient as random terms, and time, C/I ratio, loading, intermediate abutment, splinting and location as fixed factors. The normal distribution of MBLoss and MBLevel was checked using the quantile-quantile plot. A *p* < 0.05 was considered statistically significant. Statistical analysis was performed using R 4.3 (R Foundation for Statistical Computing, Vienna, Austria).

## Results

**Fig. 1:**
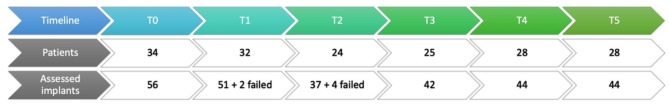
Flow diagram summarizing the number of patients and the evaluated implants at each timepoint, up to 5 years of follow-up (T5)

The analysis included 56 implants placed in 34 adults (12 males and 22 females; median age 63 years, IQR 53–66). Patient demographics and implant characteristics are summarized in Tables 1 and 2, respectively. A flow diagram summarizing the number of patients and of evaluated implants at each timepoint is presented in Fig. 1.

**Table 1:** Patient demographics

Total number of patients	34 (100%)
Male	12 (35.3%)
Female	22 (64.7%)
Age at the time of the surgery	63 (53–66)
Conventional loading*	23 (67%)
Immediate loading*	12 (35.3%)

Data summarized as n (%) or ^a^ median (IQR); * one patient received both conventional and immediate loading

**Table 2:** Summary of the collected data

Implants		56 (100%)
Location	14/24	1 (1.8%)
	34/44	2 (3.6%)
	15/25	6 (10.7%)
	35/45	4 (7.1%)
	16/26	13 (23.2%)
	36/46	14 (25%)
	17/27	7 (12.5%)
	37/47	8 (14.3%)
	18/28	1 (1.8%)
	38/48	0 (0%)
Diameter (mm) – Length (mm)	5–5	18 (32%)
	5–6	20 (37%)
	6–5	6 (10%)
	6–6	12 (21%)
Submerged healing	Yes	30 (53.5%)
	No	26 (46.5%)
Loading protocol	Conventional	40 (71%)
	Immediate	16 (29%)
Intermediate abutment (Low Profile)	Yes	18 (32%)
	No	38 (68%)
C/I ratio ^a^		2.6 (0.5) (range 1.39–3.94)
Splinted	Yes, with extra-short implant (≤ 6 mm)	27 (48%)
	Yes, with implant > 6 mm	17 (31%)
	No	12 (21%)
Cantilever	Yes	8 (14.3%)
	No	48 (85.7%)

Data summarized as n (%) or ^a^ mean (SD)

**Fig. 2:**
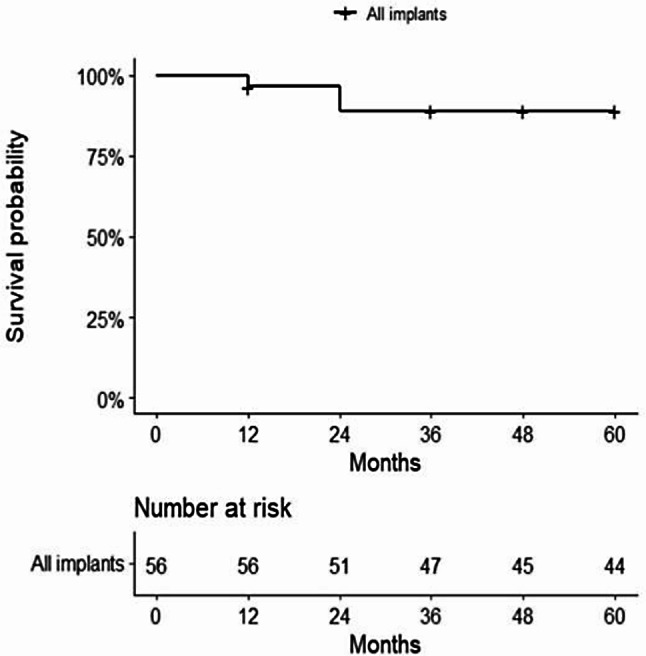
Kaplan-Meier diagram of implant survival

**Fig. 3:**
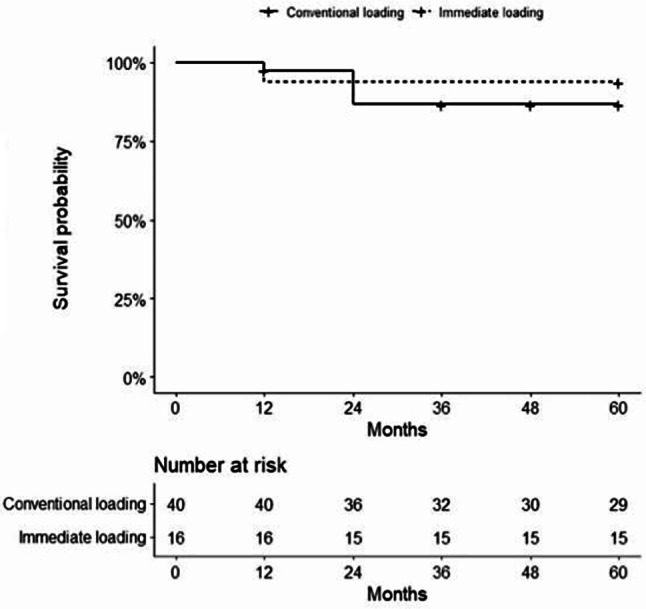
Kaplan-Meier diagram of implant survival stratified by conventional loading and immediate loading

**Fig. 4:**
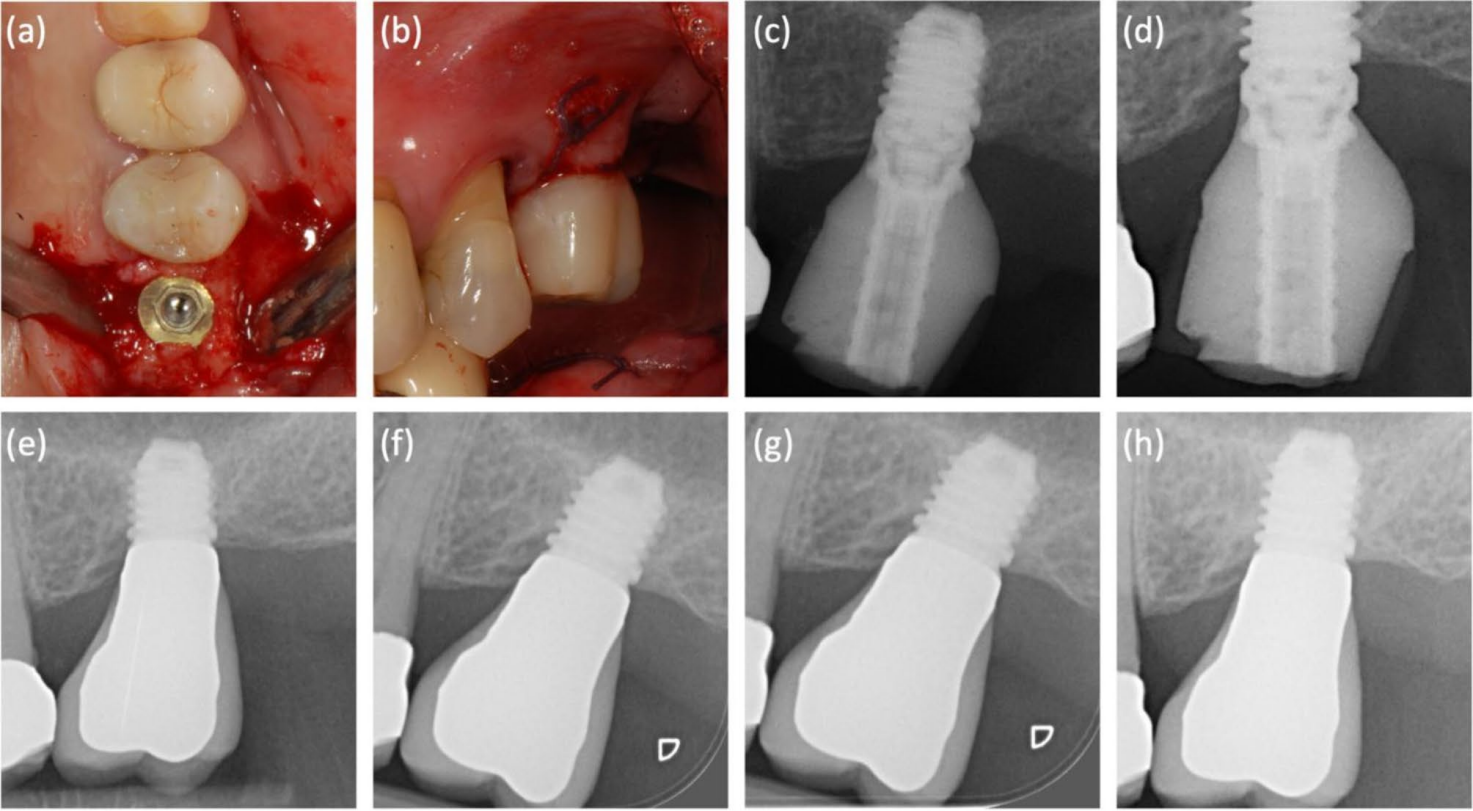
Representative case of extra-short implant (5 mm diameter and height) placed on 2.6 site with immediate non-functional loading. (**a**) Implant positioning, (**b**) immediately provisional crown, (**c**) x-ray at loading, (**d**) x-ray at 1 year, (**e**) x-ray at 2 years, (**f**) x-ray at 3 years, (**g**) x-ray at 4 years, (**h**) x-ray at 5 years

**Fig. 5:**
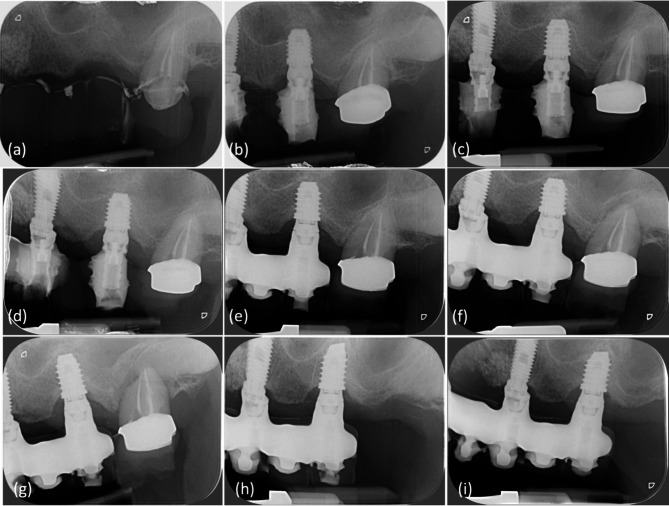
A case of full-arch immediate loading including a 5 mm diameter and 6 mm length implant placed in position 25: (**a**) preoperative periapical radiograph, (**b**) periapical radiograph taken at the time of placement and immediate loading (a 4.1 mm diameter and 3 mm height intermediate abutment was placed at implant surgery); (**c**) one-year follow-up radiograph; (**d**) two-year follow-up radiograph; (**e**) 3-year follow-up radiograph (the final restoration is in place); (**f**)-(**i**) periapical radiographs at 4–7 years of follow-up

Overall, 6 implants out of 56 failed during a median follow-up of 5 years (IQR 3–5) (Fig. 2). The implant survival was 95% at 1 year and 89% at 2–5 years (Fig. 2). Implant survival in conventional loading was 95% at 1 year and 87% at 2–5 years, while implant survival in immediate loading was 94% in 1–5 years (Fig. 3). Two representative cases are presented in Figs. 4 and 5.

Implant loss was due to peri-implantitis [38]. Two implants were lost at 1-year follow-up: the first one had been placed in the premolar region of the lower jaw, it was submerged and not splinted to other implants; the second one consisted of an immediately loaded implant placed in the molar region of the upper jaw and splinted to another implant. Both implants had no cantilever. The remaining 4 implant losses were recorded in one 52-year old systemically healthy patient with a history of periodontitis at 2-year follow-up and had been placed in molar and premolar regions of the mandible and had no cantilever. When excluding this single patient, 5-year survival was 96% in the whole sample, 97% in the group with conventional loading, and 94% in the immediate loading group.

Mean MBLoss during follow-up ranged from 0.14 mm at 1 year to 0.25 mm at 5 years (Table 3).

**Table 3:** MBLoss (mm) during follow-up

Delta T	MBLoss^§^
ΔT1-T0	0.14 (0.30)
ΔT2-T0	0.19 (0.32)
ΔT3-T0	0.21 (0.39)
ΔT4-T0	0.25 (0.48)
ΔT5-T0	0.25 (0.49)

^§^Data are expressed as mean (SD)

At univariate analysis, immediate loading was associated with higher MBLoss during follow-up (mean variation 0.21 mm, 95% confidence interval 0.01 to 0.40; *p* = 0.02) while intermediate abutment was associated with lower MBLoss during follow-up (mean variation − 0.23 mm, 95% confidence interval − 0.39 to -0.09; *p* = 0.003) (Table 4). Multivariable analysis confirmed that immediate loading was associated with higher MBLoss during follow-up (mean variation 0.21 mm, 95% confidence interval 0.01 to 0.40; *p* = 0.02) (Table 4). Intermediate abutment was not included in the model due to collinearity with loading.

**Table 4:** Investigation of factors associated with MBLoss during follow-up

	Univariate analysis		Multivariable analysis	
	β (standard error)	p-value	β (standard error)	p-value
Time	0.02 (0.01)	0.10	0.02 (0.02)	0.08
C/I ratio	0.02 (0.06)	0.73	0.01 (0.07)	0.87
Loading: immediate vs. conventional	0.21 (0.09)	0.02	0.21 (0.09)	0.02
Intermediate abutment: yes vs. no	-0.23 (0.07)	0.003	Not included due to collinearity with loading	-
Splinted with extra-short implant (≤ 6 mm) vs. non-splinted	0.17 (0.11)	0.16	0.15 (0.13)	0.26
Splinted with with implant > 6 mm vs. non-splinted	0.08 (0.11)	0.45	0.08 (0.12)	0.52
Maxilla vs. mandibula	0.12 (0.09)	0.20	0.13 (0.11)	0.24

At univariate analysis, immediate loading was associated with higher MBLevel during follow-up (mean variation 1.93 mm, 95% confidence interval 1.55 to 2.24; *p* < 0.0001) while intermediate abutment was associated with lower MBLevel during follow-up (mean variation − 1.85 mm, 95% confidence interval − 2.25 to -1.44; *p* < 0.0001) (Table 5). Multivariable analysis confirmed that immediate loading was associated with higher MBLevel during follow-up (mean variation 2.07 mm, 95% confidence interval 1.74 to 2.40; *p* < 0.001) (Table 6). In addition, MBLevel increased during follow-up (mean variation 0.04 mm, 95% confidence interval 0.01 to 0.08; *p* = 0.007) and was associate with higher C/I ratio (mean variation 0.08 mm, 95% confidence interval 0.01 to 0.06; *p* = 0.004) (Table 6). Intermediate abutment was not included in the model due to collinearity with loading.

**Table 5:** MBLevel (mm) during follow-up

Delta T	MBLevel^§^
ΔT1-T0	0.46 (0.54)
ΔT2-T0	0.63 (0.71)
ΔT3-T0	0.61 (0.76)
ΔT4-T0	0.58 (0.90)
ΔT5-T0	0.67 (0.93)

^§^Data are expressed as mean (SD)

**Table 6:** Investigation of factors associated with MBLevel during follow-up

	Univariate analysis		Multivariable analysis	
	β (standard error)	p-value	β (standard error)	p-value
Time	0.04 (0.03)	0.09	0.05 (0.02)	0.007
C/I ratio	0.08 (0.12)	0.53	0.33 (0.11)	0.004
Loading: immediate vs. conventional	1.93 (0.17)	< 0.0001	2.07 (0.16)	< 0.0001
Intermediate abutment: yes vs. no	-1.85 (0.19)	< 0.0001	Not included due to collinearity with loading	-
Splinted with extra-short implant (≤ 6 mm) vs. non-splinted	0.50 (0.24)	0.06	0.65 (0.43)	0.20
Splinted with with implant > 6 mm vs. non-splinted	0.12 (0.24)	0.61	0.19 (0.44)	0.66
Maxilla vs. mandibula	0.37 (0.21)	0.10	-0.89 (0.41)	0.07

Regarding biological complications, at one year 11 implants in 3 patients were affected by mucositis and were subjected to home and professional hygiene strengthening measures and close follow-up. Out of these, 7 cases evolved into peri-implantitis between the first and second year and to the subsequent failure of 6 implants. In one case the non-surgical peri-implantitis treatment was successful. Regarding technical/mechanical complications, 3 cases of prosthetic screw loosening without consequences (in the absence of intermediate abutment) were recorded, while no implant, screw or prosthesis fracture was observed.

## Discussion

Our study aimed to evaluate implant survival rate, MBLoss and MBLevel around extra-short implants restored with fixed prosthetic restorations. Overall, the 5-year implant survival was 89%, with lower rate reported in case of conventional loading compared to immediate loading, i.e. 87% and 94%, respectively. The mean MBLoss during follow-up ranged between 0.14 mm at 1 year to 0.25 mm at 5 years. Immediate loading was associated with a higher MBLoss during follow-up, while the presence of an intermediate abutment was associated with a lower MBLoss.

The investigation was not limited to MBLoss assessment, which was particularly relevant for the authors, because the exposure of the microrough implant surface and the progression of bone resorption could lead to a rapid implant failure in implants of short dimensions. However, since MBLoss assessment might bias the measurements in favor of the subcrestally placed implants and do not take into consideration crestal bone remodeling above the level of implant shoulder, MBLevel was calculated as well, reporting results in line with MBLoss values.

Short implants are considered a treatment option in patients with vertical atrophy of the alveolar ridge [4], likewise guided bone regeneration (GBR) [39, 40] or sinus lift [41, 42] followed by the placement of standard-length implants (i.e. length ≥10 mm). Even if they can be considered predictable procedures [43, 44], both alveolar ridge vertical augmentation and sinus lift have shown the disadvantage of significant morbidity. In the atrophic posterior mandible, vertical bone augmentation procedures are more challenging and less predictable [45, 46]. Therefore, short implants have gained popularity as an attractive alternative strategy for the rehabilitation of patients with posterior ridge atrophy.

Short implants have been demonstrated to present similar survival rates and a reduced rate of complications than longer implants placed after or simultaneously with regenerative procedures. Focusing on the rehabilitation of atrophic maxillary edentulous posterior areas, a systematic review based on eight RCTs reported lower morbidity with short implants compared with longer implants and extensive posterior maxillary grafting [5]. Fan et al. reported similar outcomes for short (5–8 mm) and long implants (> 8 mm) with sinus lifting [47]. Other systematic reviews extended their research question to the restoration of both maxilla and mandible. In another systematic review and meta-analysis short implants placed in the posterior jaws tended to exhibit a greater risk of failure compared with standard implants [48]. However, random-effect model found no statistically significant difference in survival rate between standard and short implants. A meta-analysis was also performed to evaluate whether there was a significant difference in terms of complication rate. Standard implants presented higher rates of complications by trend. Similar outcomes were found by Nisand et al. for short versus longer implants in combination with GBR [49].

A meta-analysis on the survival rates of short dental implants (≤ 6 mm) compared with implants longer than 6 mm in posterior jaw areas reported values for short implants ranging from 86.7 to 100% (from 1 to 5 years), likewise in the present report (89% survival rate at 5-year follow-up). It was concluded that short implants may be the preferable choice in some cases of maxillary and mandibular atrophy [23].

Similar survival rate (86.7%) has been registered also by Rossi et al. in a 5-year follow-up RCT in which the use of short implants for single-tooth replacement was studied [50]. However, these results do not resemble other studies on short implants, in which higher survival rates have been reported, i.e. 96.24% [49], 97.5% [51] and 95.0% [52].

Many studies reported no significant difference on marginal bone loss between short implants and long implants [15, 53] or better performances when short implants were applied [54]. In the present study, findings on mean marginal bone loss during follow-up ranged from 0.14 mm at the first year to 0.25 mm at the fifth year. These results are promising if compared to literature data. In a RCT short dental implants have been compared to long implants in combination with sinus floor elevation with a 5-year follow-up [53]. Mean MBLoss was 0.54 mm ± 0.87 (short implants group) and 0.46 mm ± 1.00 (long implants group). Rossi et al. found a mean 0.14 mm and 0.18 mm of bone loss respectively at the test (short implants) and control (longer implants) sites in a 5-year follow-up prospective multicentred RCT [50]. Gujié et al., in another 5-years follow-up RCT, found a mean marginal bone level change of 0.01 ± 0.45 mm (bone gain) in the 6 mm group and − 0.12 ± 0.93 mm (bone loss) in the 11 mm group without a significant difference between the groups [15].

Felice et al. in their RCT evaluated whether 5 mm short implants could be an alternative to augmented bone and placement of 10 mm long implants in atrophic posterior jaws [54]. Five years after loading, mandibular implants lost on average 1.72 mm (short implants group) and 2.10 mm (long implants group); on the maxillary bone, short implants lost on average 1.31 mm while long implants lost, on average, 1.79 mm. Both differences were statistically significant, demonstrating that short implants performed better. In a prospective clinical and radiographic study, Villarinho et al. reported a cumulative mean bone loss at 48 months of 0.3 ± 0.5 mm compared to prosthetic loading (baseline) [55].

According to some authors, crown-to‐implant ratio seems not to be correlated with crestal bone loss or risk of failure of short implants [56, 57]. Splinting short implants when two or more adjacent implants are present, combined with providing the patient with a mutually protected or canine guided occlusion, has been suggested to reduce the mechanical forces on individual implants and components [58, 59]. Splinting may also reduce the incidence of screw loosening/fracture, porcelain chipping, and implant overload.

Villarinho et al. showed no increase in risk of prosthetic failure for any of the potential risk factors assessed: arch, presence and intensity of bruxism, maximum bite force, anatomical C/I ratio, clinical C/I ratio, or occlusal table area [55]. However, there was a tendency toward a greater risk of failure in the mandible rather than the maxilla and for patients with bruxism. Kim and colleagues [60], in a retrospective study, observed no relationship between C/I ratio and prosthetic failure for single implants of various lengths in the posterior region, but observed a relationship between presence of mesial or distal cantilever and incidence of screw loosening. Cantilever extension was also suggested to negatively affect MBLoss over time around implants supporting 3-unit fixed partial dentures [61]. In another RCT evaluating the effect of splinting crowns in case of short implants, Al-Sawaf et al. concluded that splinting crowns on short implants seems to not affect either the amount of marginal bone loss or peri-implant health three years after loading [62].

The use of an intermediate prosthetic abutment may favor a proper healing of the supracrestal soft tissues with the consequent reduction of bone resorption given that it allows the adhesion of hemidesmosomes around the titanium with the formation of a stable biological seal [63]. Since bacterial leakage in the implant-abutment interface is considered as one the most important factor in the occurrence of inflammatory reactions around the implant, intermediate abutments can prevent bacterial infiltration, detachment of the hemidesmosomial junctions and establishment of focal inflammation [64, 65]. In the present study, application of intermediate abutments was associated with lower MBLoss during follow-up at the univariate analysis, but it could not be included in the multivariable analysis due to collinearity with loading. In a retrospective study, Galindo-Moreno et al. investigated the vertical platform-switching concept, analyzing intermediate abutment height and horizontal width in relation to MBLoss [35]. They found that horizontal width did not influence MBLoss, while implants with an intermediate abutment of 2–4 mm had less MBLoss. The same authors observed a mean MBLoss of 0.912 mm mesially and 1.182 distally at 18 months with abutments higher than 2 mm; 1.272 mesially and 1.269 distally at 18 months with abutments shorter than 2 mm. A review by Chen et al. seems to confirm the efficacy of high abutments on reducing MBLoss [36].

Several studies have showed no significant differences on marginal bone loss between immediate and conventional loading in standard length implants [66, 67].

In the present study, 16 extra-short implants were immediately loaded and the 5-year implant survival was 94%. Despite the relative low number of examined implants, this data resulted significant when compared to conventional loading in a univariate analysis. Immediate loading protocol may not increase the failure risk of short dental implants [30, 68], as demonstrated in studies utilizing single- and multiple-unit fixed dental prostheses [10, 25–29].

Our study has some limitations that should be considered. First, the retrospective design may have limited the data quality. Second, the sample size suggests caution in the interpretation of the findings. For instance, the number of implants was unbalanced according to intermediate abutment, as well as between conventional and immediate loading. Furthermore, the sample was heterogeneous in terms of some clinically relevant factors (such as implant position, splinting, loading protocol, use of intermediate abutment) but unfortunately the small sample size limited the investigation within clinically relevant strata. Finally, different prosthetic protocols were utilized. Therefore, to overcome the methodological deficiencies outlined above, a more controlled setting and a prospective study design would be desirable in future trials aiming at further exploring the effect of various factors, including immediate loading, intermediate abutment placement and cantilever extension, on the survival rate and marginal bone remodeling around extra-short implants.

In conclusion, within its limitations, this retrospective study demonstrates the clinical validity of extra-short implants, even under immediate loading conditions. The use of horizontal and vertical platform switching by means of intermediate abutments seems to be able to contribute to the reduction of marginal bone resorption, which is crucial for the survival of short length implants.

## Electronic supplementary material

Below is the link to the electronic supplementary material.

Supplementary Material 1

## Data Availability

The data that support the findings of this study are available from the corresponding author, upon reasonable request.

## Abbreviations

ASA: American Society of Anaesthesiologists
C/I: Crown/implant ratio
CBCT: Cone beam computer tomography
ICC: Intraclass correlation coefficient
IQR: Interquartile range
IS: Implant shoulder
MBLevel: Marginal bone level
MBLoss: Marginal bone loss
RCT: Randomized controlled trial
RR: Risk ratio
SD: Standard deviation
STROBE: Strengthening the Reporting of Observational studies in Epidemiology
95%CI: 95% confidence interval
