# Supplementary material for: Clinical and radiographic outcomes of extra-short implants (≤ 6 mm) in the posterior atrophic jaws: a retrospective cohort study
Source: Int J Implant Dent. 2025 Jan 20;11:4. doi: 10.1186/s40729-025-00592-z (PMC11747052; doi:10.1186/s40729-025-00592-z)
Supplement: Supplementary file 1 — Supplementary Material 1 [file 40729_2025_592_MOESM1_ESM.docx]

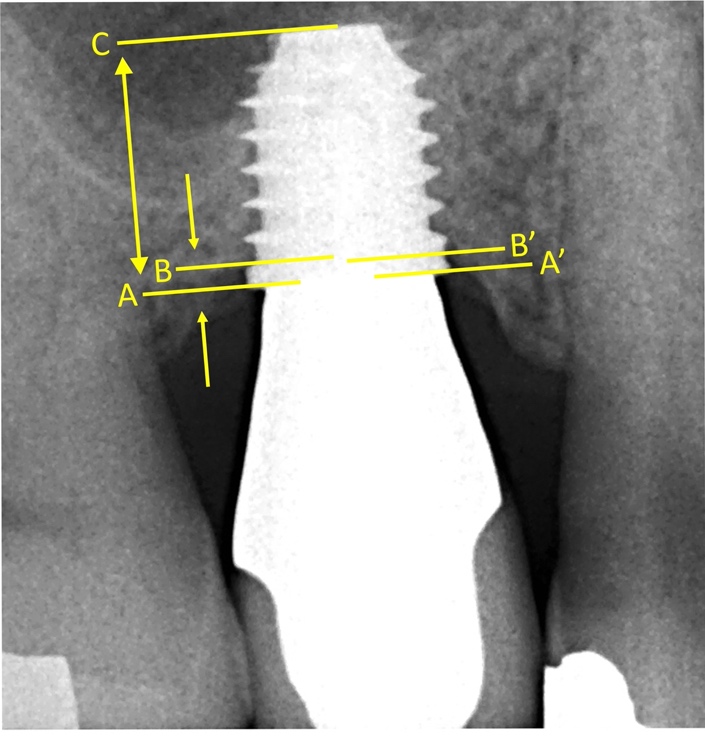


# *Supplementary figure 1. Measure of the marginal bone loss (MBLoss), obtained as the vertical distance between the implant shoulder and the most coronal bone-implant contact (A/B for distal MBLoss and A’/B’ for mesial MBLoss).*


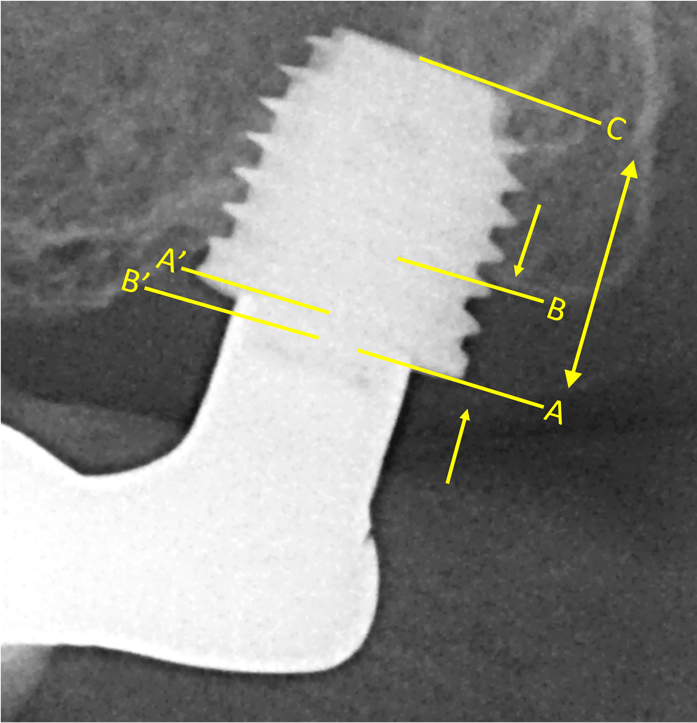


***Supplementary figure 2.*** *Measure of the marginal bone level (MBLevel), obtained as the vertical distance between the implant shoulder and the most coronal bone-implant contact (A/B for distal MBLevel and A’/B’ for mesial MBLevel). A positive value was assigned when the bone crest was coronal to the IS (A’/B’), whereas a negative value was assigned when the bone crest was apical to the IS (A/B).*
